# Supplementary material for: Assessing the impact of structural modifications in the construction of surveillance network for Peste des petits ruminants disease in Nigeria: The role of backbone and sentinel nodes
Source: PLoS One. 2024 Nov 18;19(11):e0303237. doi: 10.1371/journal.pone.0303237 (PMC11573210; doi:10.1371/journal.pone.0303237)
Supplement: S1 Table — (DOCX) [file pone.0303237.s001.docx]

*S1 Table . Number of nodes (districts) in all configurations taken as seed per States: A: reference network, B1: modified network with random reallocation of 5% of links, B2: modified network with random reallocation of 20% of links, B3: modified network*

| State | A | B1 | B2 | B3 | C1 | C2 | C3 | D1 | D2 | E1 | E2 |
| --- | --- | --- | --- | --- | --- | --- | --- | --- | --- | --- | --- |
| Plateau’s seed | 26 (41%) | 26 (41%) | 25 (40%) | 26  (41%) | 29 (39%) | 29  (33%) | 30  (29%) | 24 (40%) | 24 (39%) | 24 (42%) | 25 (43%) |
| Bauchi’s seed | 18 (29%) | 18 (29%) | 18 (29%) | 18  (29%) | 18 (27%) | 18   (20%) | 20  (20%) | 17 (28%) | 18 (29%) | 18 (32%) | 13 (22%) |
| Kano seed’s | 6 (10%) | 6 (10%) | 6 (10%) | 6  (10%) | 7   (11%) | 10   (11%) | 12  (12%) | 6 (10%) | 6 (10%) | 3 (5%) | 6 (10%) |
| Others | 13 (20%) | 13 (20%) | 14 (21%) | 14  (21%) | 15 (23%) | 31  (36%) | 40  (39%) | 13 (22%) | 14 (22%) | 9 (21%) | 14 (25%) |
| Total | 63 | 63 | 63 | 63 | 70 | 88 | 102 | 60 | 62 | 57 | 58 |
