# Supplementary material for: Assessing the impact of structural modifications in the construction of surveillance network for Peste des petits ruminants disease in Nigeria: The role of backbone and sentinel nodes
Source: PLoS One. 2024 Nov 18;19(11):e0303237. doi: 10.1371/journal.pone.0303237 (PMC11573210; doi:10.1371/journal.pone.0303237)
Supplement: S2 Table — diff: The difference between the means of pairs of cluters. lwr: Lower limit of the confidence interval for the difference. upr: Upper limit of the confidence interval for the difference. p adj: The adjusted p-value for each pair of clusters. (DOCX) [file pone.0303237.s002.docx]

***S2 Table .*** *Comparison of the average final size of the epidemic between the cluster identified by the k-means methods using Tukey test. diff: The difference between the means of pairs of cluters. lwr: Lower limit of the confidence interval for the difference. upr : Upper limit of the confidence interval for the difference. p adj: The adjusted p-value for each pair of clusters.*

| configuration | cluster | diff | lwr | uper | p adj |
| --- | --- | --- | --- | --- | --- |
| A | 2-1 | -48.55 | -49.82 | -47.28 | 0 |
|  | 3-1 | -52.19 | -53.60 | -50.79 | 0 |
|  | 3-2 | -3.64 | -4.87 | -2.41 | 0 |
| B1 | 2-1 | -12.95 | -15.14 | -10.76 | 0 |
|  | 3-1 | 32.96 | 30.78 | 35.13 | 0 |
|  | 3-2 | 45.91 | 43.73 | 48.10 | 0 |
| B2 | 2-1 | 39.13 | 36.98 | 41.29 | 0 |
|  | 3-1 | 19.40 | 17.23 | 21.56 | 0 |
|  | 3-2 | -19.73 | -21.88 | -17.59 | 0 |
| B3 | 2-1 | -38.32 | -40.53 | -36.11 | 0 |
|  | 3-1 | -48.14 | -50.37 | -45.91 | 0 |
|  | 3-2 | -9.82 | -12.05 | -7.58 | 0 |
| C1 | 2-1 | 13.39 | 10.94 | 15.84 | 0 |
|  | 3-1 | 25.72 | 23.26 | 28.17 | 0 |
|  | 3-2 | 12.32 | 9.88 | 14.76 | 0 |
| C2 | 2-1 | 129.70 | 125.69 | 133.70 | 0 |
|  | 3-1 | -7.21 | -11.31 | -3.11 | 3.56e-05 |
|  | 3-2 | -136.91 | -140.91 | -132.30 | 0 |
| C3 | 2-1 | 44.43 | 41.63 | 47.23 | 0 |
|  | 3-1 | -20.29 | -23.13 | -17.45 | 0 |
|  | 3-2 | -64.72 | -64.66 | -61.89 | 0 |
| D1 | 2-1 | 18.95 | 18.13 | 19.76 | 0 |
|  | 3-1 | 19.60 | 18.79 | 20.41 | 0 |
|  | 3-2 | 0.65 | -0.14 | 1.44 | 0.13 |
| D2 | 2-1 | 3.25 | 0.69 | 5.81 | 0.01 |
|  | 3-1 | 95.88 | 93.39 | 98.37 | 0 |
|  | 3-2 | 92.63 | 90.15 | 95.10 | 0 |
| E1 | 2-1 | -97.58 | -102.78 | -92.38 | 0 |
|  | 3-1 | -88.29 | -90.80 | -85.77 | 0 |
|  | 3-2 | 9.29 | 4.03 | 14.55 | 3.3e-05 |
| E2 | 2-1 | -59.27 | -61.10 | -57.44 | 0 |
|  | 3-1 | -67.15 | -68.99 | -65.32 | 0 |
|  | 3-2 | -7.88 | -9.76 | -6.00 | 0 |
