# Supplementary material for: Assessing the impact of structural modifications in the construction of surveillance network for Peste des petits ruminants disease in Nigeria: The role of backbone and sentinel nodes
Source: PLoS One. 2024 Nov 18;19(11):e0303237. doi: 10.1371/journal.pone.0303237 (PMC11573210; doi:10.1371/journal.pone.0303237)
Supplement: Attachment — Submitted filename: ResponseReviewers.docx [file pone.0303237.s003.docx]

We are grateful to the reviewers for their insightful comments, which have significantly enhanced the quality of our work. We read all of them thoroughly and incorporated them into our manuscript. Below are our responses and the revisions made to the article.

**Response to reviewer #1**

We thank the reviewer for all the general and specific comments. General comments gave us the opportunity to reflect and make more explicit the assumptions of our modeling approach and also to explain the definition and procedure better. We hope that the conclusions and answers provided will make the article clearer and more interesting to the reviewers and the scientific community;

**Reviewer #1: The study “Assessing the impact of structural modifications in the construction of surveillance network for transboundary animal diseases: the role of backbone and sentinel nodes” set out to identify critical districts in which to institute surveillance activities among other control activities of TADs with a special focus on Peste des petits ruminants (PPR). The study used data from 10 market surveys in 3 states and employed a mix of standard social network analysis and mathematical modelling approaches to identify the most probable “sentinel districts” in Nigeria. The authors present a strong case in regard to the urgent need to effectively control PPR and eventually eradicated through activities such as building strong and efficient surveillance systems, a key contribution to the global efforts to eradicate PPR.
However, the manuscript in current state suffers a few serious methodological and minor scholarly considerations that the authors should consider attending to so as to achieve their set objective (s).**

**General comments
The authors conveniently define ‘sentinel nodes’ as those areas that are rapidly infected first in case of disease introduction, a definition that leans more to anatomy (cancer research).**

Regarding the definition of "sentinel nodes," we understand the concern about its alignment with terminology traditionally used in anatomy, specifically in cancer research. We intend to use this term to convey the critical role these nodes play in the **early detection** and monitoring of disease spread within a network. In the context of our study, "sentinel nodes" are defined as districts (nodes in our network) that are often early infected in the event of disease introduction, thus serving as early alert points for potential outbreaks. This concept is crucial for designing and implementing an effective surveillance network, allowing timely intervention and control measures.

This article used a definition commonly applied in network literature to identify nodes for surveillance networks. For instance, Herrera *et al*. used "sensor nodes" to identify nodes likely to provide timely and accurate indications of epidemic activity. Similarly, Bajardi et *al.*, Schirdewahn *et al*., and Bai *et al*. used the term "sentinel nodes" to refer to nodes that are often infected promptly (Schirdewahn et al., and Bai et al.) and provide information on the origin of the disease (Bajardi et al.). Our definition is thus grounded in established literature, particularly the works of Herrera et al., Bajardi et al., Schirdewahn et al., and Bai et al.

We added a paragraph to relate our definition of sentinel nodes to previous work, which we inspired to

“*Previous works have attempted to identify nodes for surveillance networks. For instance, Herrera et al. used the term "sensor nodes" to identify nodes those nodes that are likely to provide timely and accurate indications of epidemic activity. Similarly, Bajardi et al., Schirdewahn et al., and Bai et al. used the term "sentinel nodes" to refer to nodes that are often infected promptly (Schirdewahn et al., and Bai et al.) and provide information on the origin of the disease (Bajardi et al.).* “

We clarified the definition of sentinel nodes in the text

*“ In this work, we defined sentinel nodes based on their vulnerability and the moment in time at which they are infected. There is no univocal definition of a node’s vulnerability. This article defines vulnerability as the probability of a node getting infected early in the epidemic. We used simulation results to identify sentinel nodes for each configuration and transmission probability. For each node, we estimated:*

- *We recorded for each simulation the time it has been infected (time of infection);*
- *We calculated the number of simulations times infection occurred before the epidemic peaked (frequency);*
- *We estimated the average frequency of infection and the standard deviation;*
- *Based on the frequency distribution, the classical threshold (average frequency + 2 standard deviations) rule was used to classify nodes;”*

*References*

Schirdewahn, F. et al. (2021) ‘Early warning of infectious disease outbreaks on cattle-transport networks’, PLOS ONE. Edited by S. Clegg, 16(1), p. e0244999. Available at: <https://doi.org/10.1371/journal.pone.0244999>.

Herrera-Diestra JL, Tildesley M, Shea K, Ferrari M. Network structure and disease risk for an endemic infectious disease [Internet]. arXiv; 2021 [cité 31 août 2022]. Disponible on: <http://arxiv.org/abs/2107.06186>

Bajardi P, Barrat A, Savini L, Colizza V. Optimizing surveillance for livestock disease spreading through animal movements. Journal of The Royal Society Interface. 7 nov 2012;9(76):2814‑25.

Bai, Y. et al. (2017) ‘Optimizing sentinel surveillance in temporal network epidemiology’, Scientific Reports, 7(1), p. 4804. Available at: https://doi.org/10.1038/s41598-017-03868-6.Bai, Y. et al. (2017) ‘Optimizing sentinel surveillance in temporal network epidemiology’, Scientific Reports, 7(1), p. 4804. Available at: <https://doi.org/10.1038/s41598-017-03868-6>.

**I would have thought that since the focus is control of TADs, a definition that is borrowed from epidemiology is more appropriate - sentinel nodes as to mimic sentinel monitoring or surveillance. These would be those districts intentionally identified to enable quick disease detection and patterns that guide more conclusive epidemiological investigations to determine spread mechanisms.**

An epidemiological approach involving intentional monitoring of districts to observe disease emergence/re-emergence over time and space could apply where logistical and financial resources permit routine data collection. However, in contexts with limited resources, such as many regions affected by TADs, implementing such comprehensive surveillance is often not feasible. In our study, we aimed to identify

critical districts for surveillance activities based on their potential to be rapidly infected in the event of disease introduction: sentinel nodes are nodes where cases could be reported before the epidemic peak (because infected animals are introduced and/or naive residents' animals are infected). This aligns with the concept of sentinel nodes in complex network and in epidemiology, as these districts serve as early warning indicators of potential outbreaks. By focusing on these districts, we can effectively deploy surveillance and control measures to mitigate the spread of diseases like PPR. In our model, we didn’t delve into the details of the transmission at the local level while mainly focusing on the spread between nodes. Because of this choice, some dynamics that are considered necessary for local transition have not been considered. Nevertheless, this approach has been widely used in Bajardi *et al.,* Schirdewahn *et al.*, and Herrera *et al.*

We explained our choices of modeling

*“The focus of this work is to study the propagation of PPR at the national level to identify possible locations where an outbreak could be reported and assess how the structure of livestock exchanges could impact the diffusion. To this end, we didn’t include livestock infection dynamics. We considered the inter-epidemic period, during which PPR is re-introduced in the area due to livestock movements from areas where cases are recorded. In the following, the term “Infected” nodes indicate nodes where new cases are registered due to the arrival of infected animals from other areas and where the transmission can occur to animals in the area. The main parameter ρ encompasses all the dynamics that could lead to the occurrence of at least a case in the area. We have omitted in this study. We have omitted several disease-related parameters that could impact the attack rate in the local population. This approach has been used in the past to identify candidate nodes for surveillance network “*

**For example districts that have a very high in-degree would be very good sentinels for disease surveillance because they simply receive animals from various origins that could potentially introduce disease.**

Surveillance of nodes with a high in-degree can be beneficial, and even nodes with high betweenness centrality can act as bridges or intermediaries in the network. However, the effectiveness of targeting high-degree or high-betweenness nodes may vary depending on the network structure and the characteristics of the disease. For instance, in the study by Colman et al.,(where the objective was to find out whether a strategy that distributes sentinel nodes in different regions of the network can be better than one that simply targets the highest degree nodes) highly modular or spatially integrated networks suggest that placing sentinels on nodes distributed across different region is preferable. Previous works by Herrera et al. have shown that indicators like eigenvector centrality are better suited to identify nodes that can be used as surveillance nodes, thus hinting at the importance of the position in the network. Furthermore, the timeliness is also related to the characteristics of the disease (in particular, the transmissibility). Because of this, in our work, we started with numerical simulations to identify sentinel nodes and, in a second step, identify the factors associated with the individual factors.

Nevertheless, estimating centrality measures on incomplete data is not always easy. Hence, our study needs to modify our networks and determine which centrality is the most reliable. Our study shows that accounting only for observed network statistics in a partially documented connection framework could be misleading. Our findings suggest a potential role of peripheral nodes in disease transmission. The increase in the size of the network also changes the infection dynamics. Therefore, the identification of consistent set of sentinel nodes regarding network modification appears essential.

**Much as the authors apply robust techniques to identify the network backbone that has high potential for surveillance activities a much clearer approach for identifying sentinels for surveillance should have considered.**

Our aim was to explore the potential utility of backbone nodes to identify sentinel candidates: we tested the hypothesis that since the backbone corresponds to an invariant set of nodes, nodes within the backbone could potentially serve as robust candidates for surveillance. To explore this hypothesis, we estimated how many sentinel nodes identified through our primary approach (nodes frequently affected before the peak) were also part of the network backbone. We found significant overlap between these sets of nodes, suggesting that nodes in the backbone may indeed be suitable candidates for surveillance.

**For example, build the network and identify communities (if they exist) and each community selected districts with the highest selected centrality measure (s) – influential nodes and then those become sentinel nodes.**

As suggested in a previous point by Colman et al., for highly modular network this approach is a valuable one. However, this type of analysis is outside the scope of the present study but we are currently working on these questions.

**Sentinel nodes would make much more sense if the authors then explored them over time (longitudinally) as cross-sectional view of the sentinels doesn’t tell a full picture.**

In our analysis, the network is considered static. One of the characteristics of “sentinel nodes” was the time of infection, which could vary depending on the characteristics of the disease, and the structure of the network. To identify the sentinel nodes we run 100 simulations for each combination of probability and network configuration and recorded if and when a node was infected before or after the peak. The distribution of the time of the infection, was used to identify sentinel nodes.

Unfortunately, we didn’t have the possibility to explore the sentinel nodes over a temporal network since no data on network evolution were available. This point was raised in the discussion.

*“As the quality of the prediction depends on the epidemic parameters, such as transmission and recovery rates, a more sophisticated model (SIR) would be capable of better capturing the interplay of animal movements (structure, volume, and* ***temporal aspects****) and transmission dynamic”*

**The authors need to explain more about their choice of approach to deal with missing/unreliable survey data especially inline with the natural network structure of livestock mobility in Nigeria**

As in many sub-Saharan African countries, Nigeria doesn’t have a system to collect and map livestock movements, making it challenging to define a natural network. The work conducted by the Lidiksi project and presented in this article represents the first case where livestock mobility network has been reconstructed. However, since the survey is not exhaustive, we need to explore scenarios where further movements (corresponding to links) exist but have not been recorded, as well as the effect of indicating/misplacing locations of orgin or destiantion of movements. In most cases, these limitations could result in a network with less or re-oriented links. Considering all the possible network configurations will require exploring a very large space of configurations and the interpretation of the results would be difficult.

We explained this in the text:

*“Various errors can generally be encountered during surveys. In our case, measurement errors were possible because incorrect responses can be given (e.g., village names may have the same name or be mispronounced, leading the interviewer to record a wrong name). Another potential error was sampling error; the sample of interviewees (100 respondents) might not represent all traders passing through the markets where the survey is conducted. We assume the reference network (configuration A) is merely represent only a subset of the “actual” animal mobility network, and several movements could not be recorded during the activity (non-observed links) or recorded inappropriately. Our analysis inferred the role of non-observed links and network modifications in disease propagation and the identification of sentinel nodes” also here “Modifications due to structural misinformation: For several reasons respondents could be referring to misleading origins/destinations of movements. Among these reasons, we could cite the difference among official name and commonly-used name for identify location, the absence of an official database of villages, but also the reticence of the respondent”*

**In the epidemic simulation, the authors chose a “district” as the unit to be susceptible or “infected” and simulated the speed and breadth of PPR penetration across the different nodes. The authors need to elaborate more why they ignored other important epidemiological parameters that affect PPR transmission such as the small ruminant population density in each district, vaccination coverage, PPRV “spreadablity” (R0), animals that recover may not be “infectious” in the next cycle of infection etc. A suitable explanation needs to be included such that this study is more reliable.**

The aim of our work is to understand the spread of PPR at the regional scale in order to identify critical sentinel nodes for surveillance. Becuase of this distric were used as nodes and we didn’t study the transmission within district popualation.In that view, movement data were collected in markets of different states and used to simulate the spread at a regional scale. The paper's take-home message is to pinpoint the characteristics of sentinel nodes and their role in the network. Assuming an SI model might be a rough hypothesis, but it makes sense that it works at the market level, not the animal.

To make it clearer, we have added, we add in Method this sentence:

*« The focus of this work is to study the propagation of PPR at the national level to identify possible locations where an outbreak could be reported and assess how the structure of livestock exchanges could impact the diffusion. To this end, we didn’t include livestock infection dynamics. We considered the inter-epidemic period, during which PPR is re-introduced in the area due to livestock movements from areas where cases are recorded. In the following, the term “Infected” nodes indicate nodes where new cases are registered due to the arrival of infected animals from other areas and where the transmission can occur to animals in the area. The main parameter ρ encompasses all the dynamics that could lead to at least a case in the area. We have omitted several disease-related parameters that could impact the attack rate in the local population. This type of approach has been used in the past to identify candidate nodes for surveillance network »*

**Specific comments
The title needs to be revised to include PPR or Nigeria (or both). Or if the title will remain as is, the introduction section of the abstract needs to start off with more general literature about livestock mobility, sentinel nodes etc as PPR seems sneaked in the introductory paragraph of the abstract (given that it is not anywhere in the title).**

We agreed and changed the title to :

*« Assessing the impact of structural modifications in the construction of
surveillance network for Peste des petits ruminants in Nigeria: the role of backbone and sentinel nodes »*

**Lines 6—10: Please crosscheck with the journal guidelines on author affiliation addresses otherwise in their current form they are abbreviated to the extent that one can hardly figure them out**

The guideline was checked and corrected

**Line 15: please rectify this ‘sweeping’ statement “....and is transmitted through livestock movements” because it not entirely true**

The sentence in Line 15 has been changed accordingly to the suggestion and now reads as:

*“Livestock movements contribute to the spread of the disease by introducing it to naive areas or exposing susceptible animals to it in infected regions”*

Line 20: rework that sentence as it is difficult to comprehend

The sentence has been rewritten and now read as:
 *“Using data from a market survey conducted in Northern Nigeria, we reconstructed the small ruminant’s mobility network and simulated the diffusion of the PPR virus through animal movement. From the analysis of simulation outcomes, we investigated which nodes could act as sentinel nodes under specific conditions on disease transmission.”*

Line 42: a lot of general information is included here without supporting reference. For example, lack of storage facilities – the authors need to state what that means in an ambiguous manner, do you mean fridges/freezers, warehouses?

We agree with the interpretatin ofthe reviewer.A reference has been added together with a list of specific facilities. The sentence now reads as:

*“Due to a lack of infrastructure and storage facilities (like slaughterhouses, fridge cells and warehouses, high speed road and adapted trucks), live animals are sold at the nearest local market and then moved through the commercial chain toward larger metropolitan areas for consumption (1). “*

**Line 47: please consider introducing the abbreviations in full and then only use them on subsequent mentions. For example, what does “WHOA” stand for? Additionally, thrught the entire manuscript, please write Peste des Petits Ruminants as sentence case (only capitalise the first letter), as it is just a name (noun) of a disease, so only the first letter should be capitalised**

A misspelling has been made, and WHOA stands for WOAH (World Organisation for Animal Health). The sentence has been corrected ( and WOAH changed all along the article):

*“Among these animal threats, Food and Agriculture Organisation (FAO) and World Organization for Animal Health (WOAH)have targeted Peste des petits ruminants (PPR) for eradication by 2030. “*

**Line 49: high morbidity and mortality rates: mention some figures and give credit to the sources (as “high rates” does not communicate much)**

Estimates for morbidity and mortality from previous study and WOAH estimates has been added. The sentence now reads as : *“The disease mainly affects small domestic ruminants (goats and sheep) and, due to the high morbidity and mortality rates (In the epidemic situations, PPR-related morbidity is as high as 90-100 %, with a mortality rate that can be in the range 50-90 %$ (WOAH), whilst in endemic situations, mortality can be around 33-$37 % involved, which is one of the obstacles to the economic development of affected countries.”*

**Line 50: The PPR eradication strategy actually stresses vaccination as the main-stay control intervention. Consider adding a sentence to this paragraph to give non specialist readers more context about the eradication plan.**

We agree with the reviewer that PPR strategy stresses on vaccination. But surveillance is also included and should be improved. More details on the Global Eradication and Control strategy have been provided and a new sentence added:

*“The PPR Global Eradication and Control Strategy (GCES) is composed of 4 main stages to assess the country's status and activities to be implemented to achieve the status of a PPR-free country. Vaccination is considered the main control method; however, strengthening the surveillance system is also crucial to achieving eradication. In particular, as an output of stages 3 and 4, surveillance system should be adapted for early warning (stage 3) and focused on the population at risk (stage 4)”*

**Line 109: This is the first figure, but it labelled figure 8. The figure numbers need to be harmonised throughout the write up. And what state is represented by color grey? Please rework the figure legend**

The grey areas indicate state that are not include in the study. The figure legend has been modified to include the gray color and its meaning.

**Line 111: please state clearly how many farmers exactly were interviewed per market and was this survey conducted in the same month, repeated observations or just one-off etc? Also consider replaced “were questioned” with “interviewed”**

Few information about the data collection procedure were added, in particular the number of interviewees by market (around 100 per market), and the frequency of the market survey. However, since more information will be provided about the procedure , choice of respondents and the protocol, in a paper by Sandra Ijoma, we’d like not to provide too many information at this stage. The sentence now reads as:

*“The choice of the markets was based on the outcome of a workshop with stakeholder ( Ijioma under submission). In each market, around 100 livestock owners/ traders were interviewed about the origin/destination of the movements (State, LGA, District, and name of the village), the number of animals of each species, and the reason for the movement. Each market was visited by Dr Ijioma and local enumerators once during the period between April and September 2022 (one-off). In parallel to the market surveys, focus group discussions were held with actors to complement information. However, this last set of data was not included in our analysis but is the object of a separate work (Ijioma , under submission).”*

**Line 114: “and to avoid problems related to the misspelling of village names” , the authors need to explain why they were unable to recruit local enumerators who could correctly spell the names of villages. Otherwise, this sounds like no one can correctly spell names of villages in Nigeria**

As mentioned earlier in a general comment, local enumerators were recruited for this work. However, they were confronted to some problems when recording the information: official names and commonly used names could be different; there is no unique way of transcribing location name; localities belonging to the same district/ward could be given the same name; and finally, because of the previous points, geolocalising villages was difficult, and a geodatabase of villages in Nigeria is not yet available. Because of this, we aggregated data at a higher level. This part has been reworked as:

*“Locations recorded during the survey were georeferenced. However, the identification of the villages was difficult because of different factors, among them, the absence of an official database of villages in the area to refer to also for the transcription, the difference between official village names and commonly-used ones; the use of referring to the district to indicate locations. Because of these issues, data were aggregated at the district level (network nodes). ”*

**Line 119: the authors need to include an explanation for their assumption of district susceptibility without considering vaccination coverage, animal population density, recovery from PPR confers protection (and animals may or may not be infectious to others etc). Why these assumptions were not included in the model needs to be explained**

Weagree with the reviewer that a more realistic model should incorporate additional parameters such as vaccination coverage, animal population, and recovery, considering that animals can recover from PPR and develop lifelong immunity. This was highlighted as a limitation/perspective in the discussion.

**"As the quality of the prediction depends on the epidemic parameters, such as transmission and recovery rates, a more sophisticated model (SIR) would be capable of better capturing the interplay of animal movements (structure, volume, and temporal aspects) and transmission dynamic”**

However, the aim of the work is to understand the spread of PPR at the national scale in order to identify critical sentinel nodes for surveillance. Because of this, we simplified the dynamics and didn’t include them within livestock infection dynamics to highlight the role of the structure. At the same time, the definition of an infected node takes a different meaning. In this work, an Infected node corresponds to a node where at least a new case is registered following the introduction of an infected animal from another area.

*“The focus of this work is to study the propagation of PPR at the national level to identify possible locations where an outbreak could be reported and assess how the structure of livestock exchanges could impact the diffusion. To this end, we didn’t include within livestock infection dynamics. In Nigeria, PPR is endemic with seasonal outbreaks. PPR prevalence varies widely in the countries from 40% to 11% and the vaccination coverage rate is still low. We considered the inter-epidemic period, during which PPR is re-introduced in the area, due to livestock movement. In the following, the term “Infected” nodes indicates, nodes where new cases are registered due to the arrival of infected animals from other areas and where transmission occur to animals in the area. The main parameter ρ encompasses all the dynamics that could lead to the occurrence of at least a case in the area. We have omitted in this study. We have omitted several disease-related parameters that could impact the attack rate in the local population. This approach has been used in the past to identify candidate nodes for surveillance network”*

Line 141: more elaboration is needed on “non-observed links”, what are they and how can they be identified without necessarily observing them

We identified with non-observed link the set of movements that could not be recorded during market surveys. In fact, only 100 of traders in the market could be interviewed and other movements couldn’t be collected. We rewrite the part including Line 147

*“ Since market surveys weren’t exhaustive, i.e. only a fraction of traders in the market were interviewed, We assume the reference network (configuration A) is merely represent only a subset of the “actual” animal mobility network, and several movements could not be recorded during the activity (non-observed links). Our analysis inferred the role of non-observed links and network modifications in disease propagation and the identification of sentinel nodes”*

**Line 147: the sentence is vague. Consider re-writing it in a more concise manner**
See previous answer

**Line 151: how was ‘misinformation’ judged? What gold standard did the author use to determine that the responses from the survey contained “inaccurate information”.**

There is not a golden standard to measure misinformation since no centralized system has been put in place. To our knowledge, these data are the only ones available for reconstructing mobility network. However, bias arose during the survey, as shown in some qualitative research articles. Unfortunately, we cannot quantify their effect, and the only option is to explore some scenarios to test the impact of other links on the disease's diffusion and identification of the sentinel nodes.

**This needs to be made clear in the methodology.**

**Again, state how many farmers (individual or groups of farmers) were interviewed as opposed to stating that “only some of the farmers”**

We modified the sentence that now reads as “around 100 traders in each market were surveyed.”

**Random permutations do not seem to mimic natural small ruminant movement network structures. For example randomly re-ordering links may not necessarily mimic how naturally the “sources of animals eg rural areas” to “urban destination because that is where demand for livestock and their product is” – may not be a scenario one can just assume that the direction of flow can randomly be reversed.**

We agree with the reviewer that the re-ordering doesn't natural small ruminant data. In this article, we didn’t model the network structures. However, we permuted in such a way that the indegree of a node remains fixed, thus corresponding to a permutation of the origin of the movements while maintaining fixed the destination. Because of this, the network is still formed by the links between “sources” and “destinations” , cahnging the identities of the origin without changing the direction. In this way , destination nodes are exposed to different areas although maintinag the same level of indegree.

A sentence has been added and now it reads as

*“We randomly reordered 5% (configuration B1), 20% (configuration B2), and 40% (configuration B3) of network links while maintaining the same node indegree for destination locations. This situation corresponds to the case when inaccurate information about origin of the movements are provided.”*

**In my opinion, in addition to all the innovative approaches employed by the authors to correct for missing/misleading information:
The authors needed to use their market survey data and triangulate it with local key informants with subject specific knowledge to validate the reposes from the surveys. Additionally, the authors should have explored other mechanisms of data collection such as participatory mapping exercises with key stakeholders to reconstruct reliable and robust movement networks that are closer to the true picture in Nigeria (as has previously done by other researchers eg , ). Otherwise, the authors need to clearly state the epidemiological assumptions behind their choice of correction of missing data**

In the material and Methods, data and epidemic simulation part, a section about the procedure used and the other activities are done to collect and triangulate data was added

*“The choice of the markets was based on the outcome of a workshop with stakeholder ( Ijioma under submission).In each market, around 100 livestock owners/ traders were interviewed about the origin/destination of the movements (State, LGA, District, and name of the village), the number of animals of each species, and the reason for the movement. Each market was visited by Dr Ijioma and local enumerators once during the period between April and September 2022 (one-off). In parallel to the market surveys, focus group discussions were held with actors to complement information. However, this last set of data was not included in our analysis but is the object of a separate work (Ijioma , under submission). Locations recorded during the survey were georeferenced,. However, the identification of the villages was difficult because of different factors, among them, the absence of an official database of villages in the area to refer to also for the transcription; the difference between official village name and commonly-used one; the use of referring to the district to indicate locations. “*

**Line 177: Once again it should be stated whether ‘frequency of infection’ takes care of the fact that infected animals in one epidemic may not be “infected again” as those animals will no longer be susceptible and thus move into the next class of “recovered”. It is not clear if such assumptions were considered in the simulation exercises. Additionally, for districts with not-large-enough populations of small ruminants, the epidemic will naturally die out once susceptible individuals are exhausted, and therefore I would expect some consideration of small ruminant density as a parameter. The authors need to provide an explanation or at least acknowledge that they are aware**

As previously stated we have focused only on propagation of PPR at national level and because of his we haven’t considered the population in the node. In our model, nodes are either infectious (meaning at least an outbreak is reported) or susceptible The frequency of infection in our model is considered as the number of times a node is infected among the different simulations with the same set of parameters. at each simulation the initial conditions are applied and all the nodes are susceptible, except one

We define as frequency of infection as the number of times, among all the simulations a node is infected.

**Line 231: why did the authors expect the characteristics of nodes (locations eg districts) to change with increasing probability of infection?**

Characteristics like geographical position don’t change with probability. However, we supposed and tested that the vulnerability of a node could change depending on the severity and rapidity of the disease. Vulnerability depends not only on individual characteristics but also on its position in the network and time frame. Because of this, a non-linear relation should be expected between the probability of infection and position in the network

**And also because some nodes were removed, edges re-wired in the different configurations, some changes were really expected even before the analysis.**

Changes were expected, however the magnitude of these changes couldn’t be predicted a priori and depend on the actual modification

**The question is; how do these configurations mimic the natural network structure of small ruminant movement in Nigeria (both static and dynamic).**

In our article, we considered only static network, since not enough information were available for time changes. The idea of the paper is to assess how different could be the outcomes of simulation depending on the degree of knowledge of the structures and advocate veterinary Services to collect information about missing data

**Line 286: figure legend needs to be rewritten so as to clearly communicate what the fiure represents:**

The legend has been changed to

*Results of PPR simulations using the SI model, showcasing the animal mobility network. The average final size in simulated epidemics is presented under different configurations (from A to D2) and for each transmission probability (ρ = 0.01, 0.05, 0.1, 0.15, 0.2, 0.25, 0.3, 0.5, 0.75). The final size fluctuates significantly under condition C3 (when adding 40% of links) and under condition D1 (when deleting the most centrally connected node)*

**Lines 354: The manuscript is silent about “Data analysis” section eg how the analyses were done, what software tools etc. In addition to the random forest plots, consider using the effect size and then explore visualizing the plots with interpretation of what metrics has the largest or smallest effect sizes (a suggestion)**

A sentence has been added All analyses were performed using R software (version 4.3.3) and the following packages: ggplot2, igraph, and randomForest. We did’t retain useful for this article to do an effect size analysis

**Answer to reviewer #2**

We thank the reviewer for the enthusiastic feedback. We included all suggestions in our final version. In particular, we highlighted the positive impact that targeted surveillance can have

**Reviewer #2: Excellent piece of research. - well described methods and results. Noting i am not a high level expert in network analysis this was helpful!**

**i would like to see some inclusion of the application in the discussion.**

**Sentinel surveillance is cost-effective and if targeted appropriately contributes very relevant data to overall disease control Could tools be developed to make this sustainable for example?**

We agree with the suggestion and we added in the discussion.
 *“Targeting nodes with high in-degree and in-H index can make PPR sentinel surveillance cost-effective by concentrating resources on this critical area that are more likely to detect disease timely points likely to facilitate disease spread. The outcomes of this work would help focus surveillance efforts where they are most needed, optimizing the use of resources and reducing the costs associated with widespread monitoring. To implement it and made it sustainable To make this sustainable, several strategies can be employed: redistributing veterinary officers and/or CAHWs (Community Animal Health Workers) in these sentinel areas to report routine information about the epidemiological situation; developing automated data collection platforms fed by the veterinarian officers that could provide an alert on real-time to streamline operations; involving communities through engagement and training programs to enhance local participation and reporting; developing automated data collection platforms fed by the veterinarian officers that could provide alerts on real time to streamline operations. These approaches would help focusing surveillance efforts where they are most needed, optimizing the use of resources and reducing the costs associated with widespread monitoring”*

**I was not able to access a key reference - no.15 to ensure complementarity rather than duplication/permissible overlap.**

This article is an invited one, and an extension of reference 15. The editors of the special edition will compare the two versions to assure the absence of overlap and duplication. We added a copy of the reference in the github associated to the paper (https://forgemia.inra.fr/umr-astre/manuscripts/mobility-and-ppr-diffusion-in-nigeria/-/tree/main/Papers)

**Some minor suggestions/edits:
L31 45% 'of the' sentinel of sentinel node
L40 "pillar" .. Better 'essential' or similar wording -**

The sentence has been changed accordingl*y “ In sub-Saharan Africa, livestock mobility is essential to the production”*

**L45 "unify a region" unclear –**

The sentence has been changed to - *“These movements are in most of the cases international and concentrated between two areas : the arid gridlocked areas , where most of the livestock is concentrated and to humid and greener areas of the West Africa region where most of the population lives. Transhumance and commercial livestock movements for trade can unify a region and provide red meat for the population while facilitating the spread of animal and zoonotic diseases “*

**L37 WHOA ---**This word have been changed to “WOAH”

**L51 "massively" -** suggest 'extensively' The word is maintained “massively”

L60 data's This word have been changed to “data”

**L68 "and circulating" not needed**

This word have been deleted : “In Nigeria, PPR is enzootic, with seasonal outbreaks mainly…”

L73 "of PPR" …The word “virus” have been added `PPR virus'

**L74 "line" to**

This word have been corrected to 'lineage

**L77 'ad hoc' (italics)**

**T**his word have been corrected and written in italics '*ad hoc’*

**L88 "and if so" – doesn’t read well - delete or reformat sentence.**

“and if so” have been deleted and the sentence reads as

“We checked under which network conditions and transmission probability”

**L147 "tantamount" - paramount / supreme / substantial**

The sentence have been rewrite “could demand a substantial effort”

**L286 Fig 2 caption on different page to figure.**

The caption of the figure has been modified and put in order

**L295 Fig 3 caption on different page to figure**

The caption of the figure has been modified and put in order

**L355 Gini T**

he word has been corrected to “Gini”

References
**Ref 2 "bavf"?**

The reference hasbeen corrected Diallo, A. (2008) ‘LA PESTE DES PETITS RUMINANTS : UNE MALADIE LONGTEMPS IGNORÉE’, Bulletin de l’Académie vétérinaire de France, (1), p. 273. Available at: <https://doi.org/10.4267/2042/47951.Dia>

**Duplicate refs 30/31**

The reference has been deleted

**Replace in english for "disponible sur.." and names of months**

Disponible has been changed to available and names of the month changed to english one
